# Facial Abnormalities Following Warfarin Exposure In Utero

**DOI:** 10.7759/cureus.93137

**Published:** 2025-09-24

**Authors:** Sharon Nyesiga, Happy L Mbabazi, Samalie M Kitooleko, Emmy Okello, Juliet Nabbaale

**Affiliations:** 1 Adult Cardiology, Uganda Heart Institute, Kampala, UGA; 2 Nursing, Uganda Heart Institute, Kampala, UGA

**Keywords:** anticoagulation during pregnancy, mechanical valves, rheumatic heart disease, teratogenicity, warfarin

## Abstract

In low- and middle-income countries like Uganda, rheumatic heart disease (RHD) remains a leading indication for valve replacement, disproportionately affecting young women of reproductive age. Pregnancy in women with mechanical heart valves (MHVs) poses significant challenges due to the need for anticoagulation therapy, balancing maternal thrombotic risk against fetal safety. Warfarin, though highly effective in preventing valve thrombosis, carries teratogenic risks, including warfarin embryopathy, particularly when used during the first trimester.

We report a case of a 21-year-old woman with RHD, who underwent mitral valve replacement with a mechanical prosthesis in 2021. She was poorly compliant with follow-up appointments, had a low time in the therapeutic range of 60% (target is at least 70%), and had thrombotic complications, including prosthetic valve thrombosis and an ischemic stroke. Her pregnancy was discovered at 16 weeks gestation, well beyond the critical embryogenesis period. She remained on warfarin throughout pregnancy until 36 weeks, when she was switched to low-molecular-weight heparin before delivery by elective caesarean section. A baby girl was born with features consistent with warfarin embryopathy, including saddle nose deformity, respiratory distress, and congenital heart defects.

This case underscores the teratogenic risks of warfarin and highlights the challenges of anticoagulation management in pregnant women with MHVs. While warfarin remains the most effective anticoagulant for maternal safety, its fetal risks necessitate individualized anticoagulation strategies. The case reinforces the importance of preconception counselling, contraception planning, and shared decision-making regarding valve selection and anticoagulation in women of reproductive age with MHVs. Further research is needed to optimize anticoagulation management, particularly in developing countries where RHD remains a major burden.

## Introduction

In high-income countries, degenerative valve disease in older individuals is the leading cause of valve replacement [1]. In contrast, in low- and middle-income countries such as Uganda, rheumatic heart disease (RHD) remains the predominant aetiology often affecting young individuals, including women of reproductive age [2]. The 2021 European Society of Cardiology/ European Association for Cardiothoracic Surgery guidelines for the management of valvular heart disease state that a bioprosthetic valve should be considered in young women contemplating pregnancy [3]. However, bioprosthetic valves carry a high risk of structural valve degeneration and the potential need for re-operation, whereas mechanical valves offer greater durability but require lifelong anticoagulation [4]. In pregnant women with a mechanical heart valve (MHV), determining the optimal anticoagulation regimen is challenging, as all available options pose varying risks to both the mother and the foetus [5]. Warfarin is the most effective anticoagulant for preventing thrombosis in patients with MHVs. However, warfarin easily crosses the placenta due to its low molecular weight, and multiple adverse fetal outcomes have been documented [6].

This case report describes a rare presentation of warfarin embryopathy and adds to the existing literature on its clinical manifestations and management strategies.

## Case presentation

**Figure 1:**
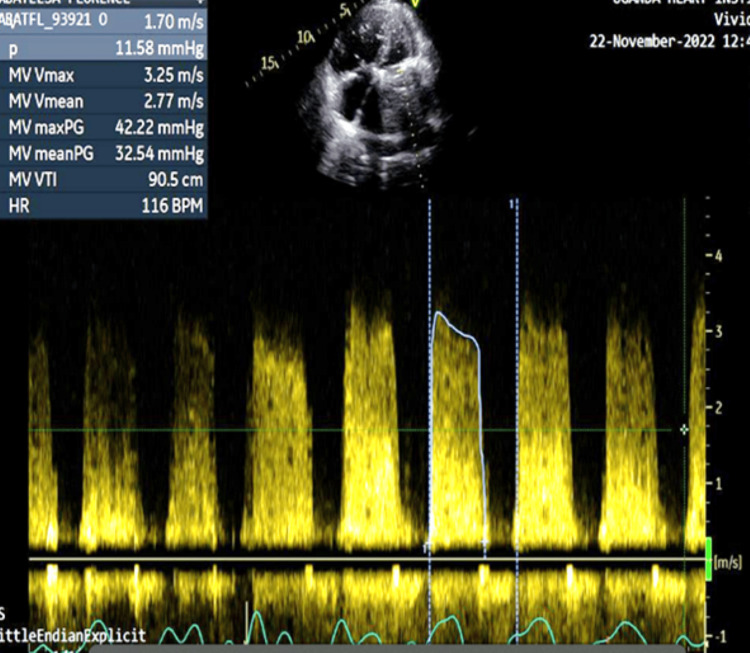
Spectral Doppler across the mitral prosthesis shows markedly elevated gradients

We present a case of a baby girl born to a 21-year-old woman who was initially diagnosed with RHD in 2018 at 14 years of age, presenting with severe mitral regurgitation (MR) and severe tricuspid regurgitation (TR). Approximately three years later, in 2021, she underwent mitral valve replacement (MVR) with a mechanical prosthesis and tricuspid Valve repair. Her immediate post-operative course was uneventful, and she was discharged on oral Warfarin (target INR 2.5-3.5), aspirin, furosemide, bisoprolol and ramipril. However, adherence to follow-up visits was poor, resulting in a suboptimal time in the therapeutic range of 60% (target =/>70%). She cited financial challenges as the cause. In November 2022, about one year after the valve replacement, she was hospitalized in cardiogenic shock. At that time, her INR was 1.3 (target INR 2.5-3.5) and transthoracic echocardiography was consistent with prosthetic valve thrombosis with markedly elevated flow gradients across the mitral prosthesis, as shown in Video 1 and Figure 1.

**Video 1:** Parasternal long axis view showing a mass (thrombus) attached to the atrial side of the mitral prosthesis

**Figure 2:**
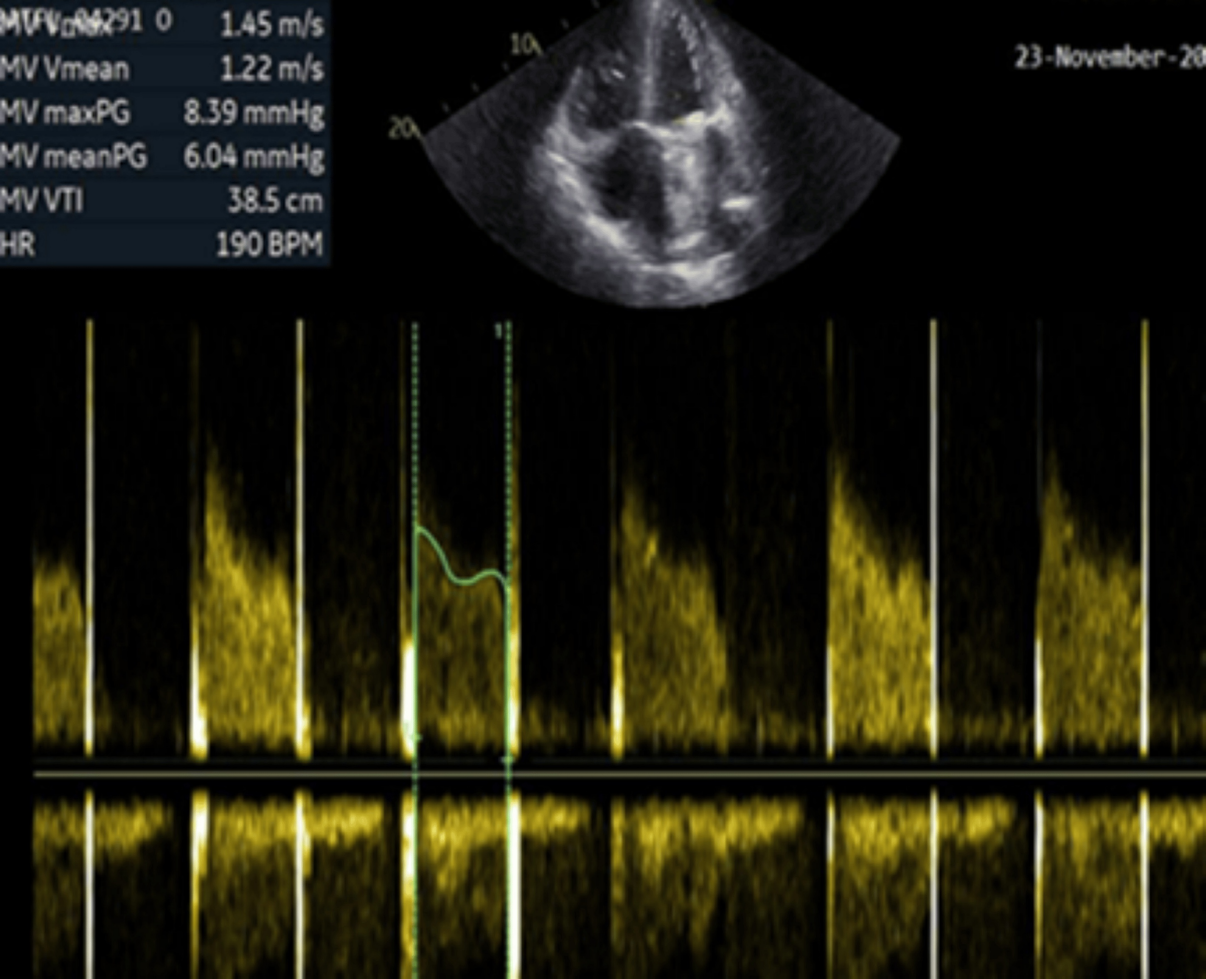
Spectral Doppler across the mitral prosthesis post lytic therapy

She was managed with systemic thrombolysis using intravenous Tenecteplase and received supportive care, resulting in a good outcome. Following the lytic therapy, the transvalvular mean pressure gradient decreased from 32 mmHg to 6 mmHg as shown in Figure 2.

**Figure 3:**
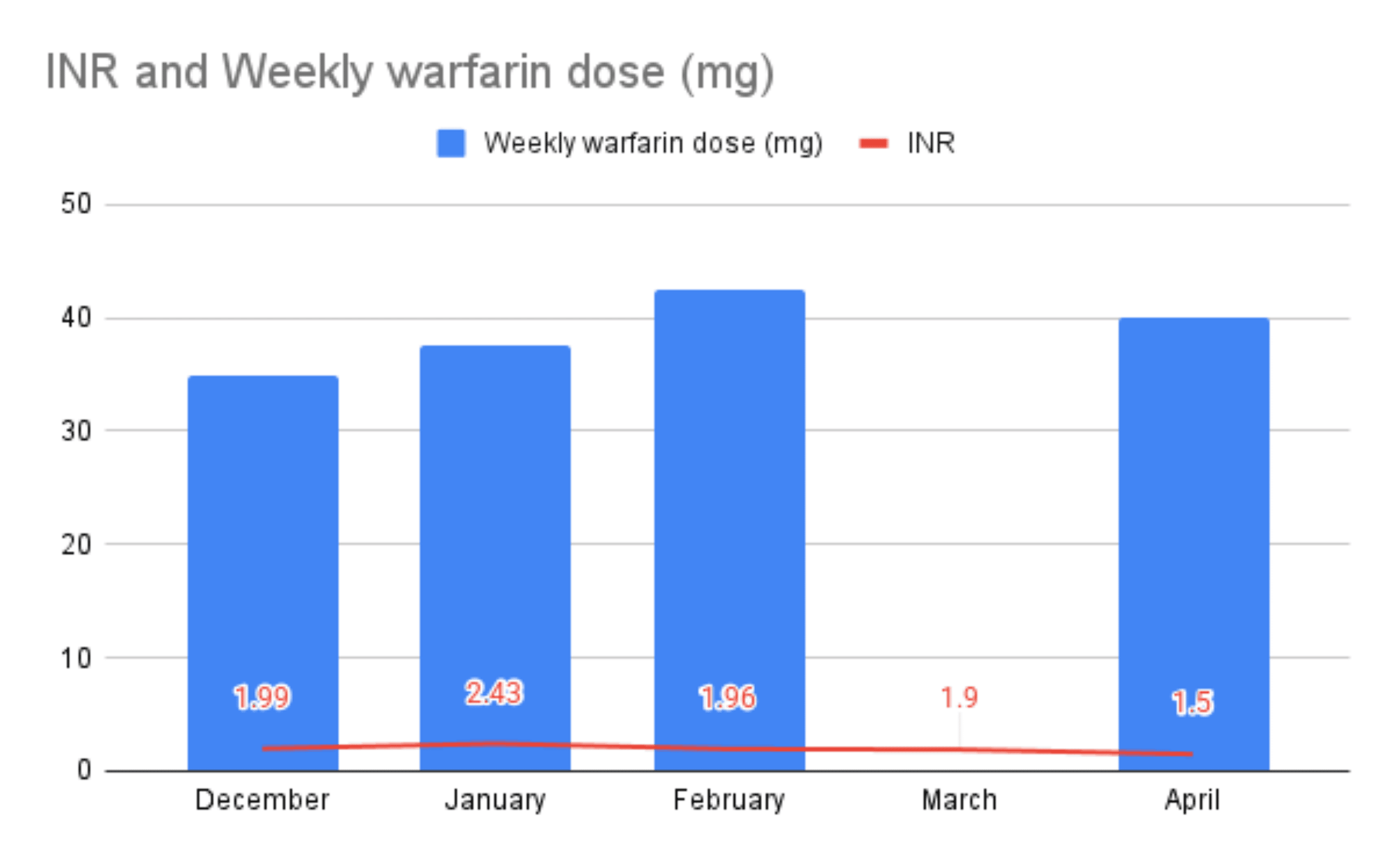
Average monthly warfarin dose and INR values during the first trimester INR: International Normalized Ratio

She was admitted again approximately one year later, in January 2024, following a history of a fall, aphasia, and right-sided weakness. At that time, she was diagnosed with an ischemic stroke. Her INR was 0.9. She was managed conservatively and subsequently discharged home. Two months after this event, she was found to be pregnant at approximately 16 weeks of amenorrhea. Her INR monitoring and warfarin dosing over the preceding four months are shown in Figure 3.

The maternal clinical course was uneventful, with continued oral warfarin therapy and regular follow-up by both cardiology and obstetrics teams. Anticoagulation was switched to low-molecular-weight heparin (LMWH) at 36 weeks of gestation, and delivery was by elective caesarean section two weeks later, in September 2024. No maternal complications, such as postpartum hemorrhage, were reported.

**Figure 4:**
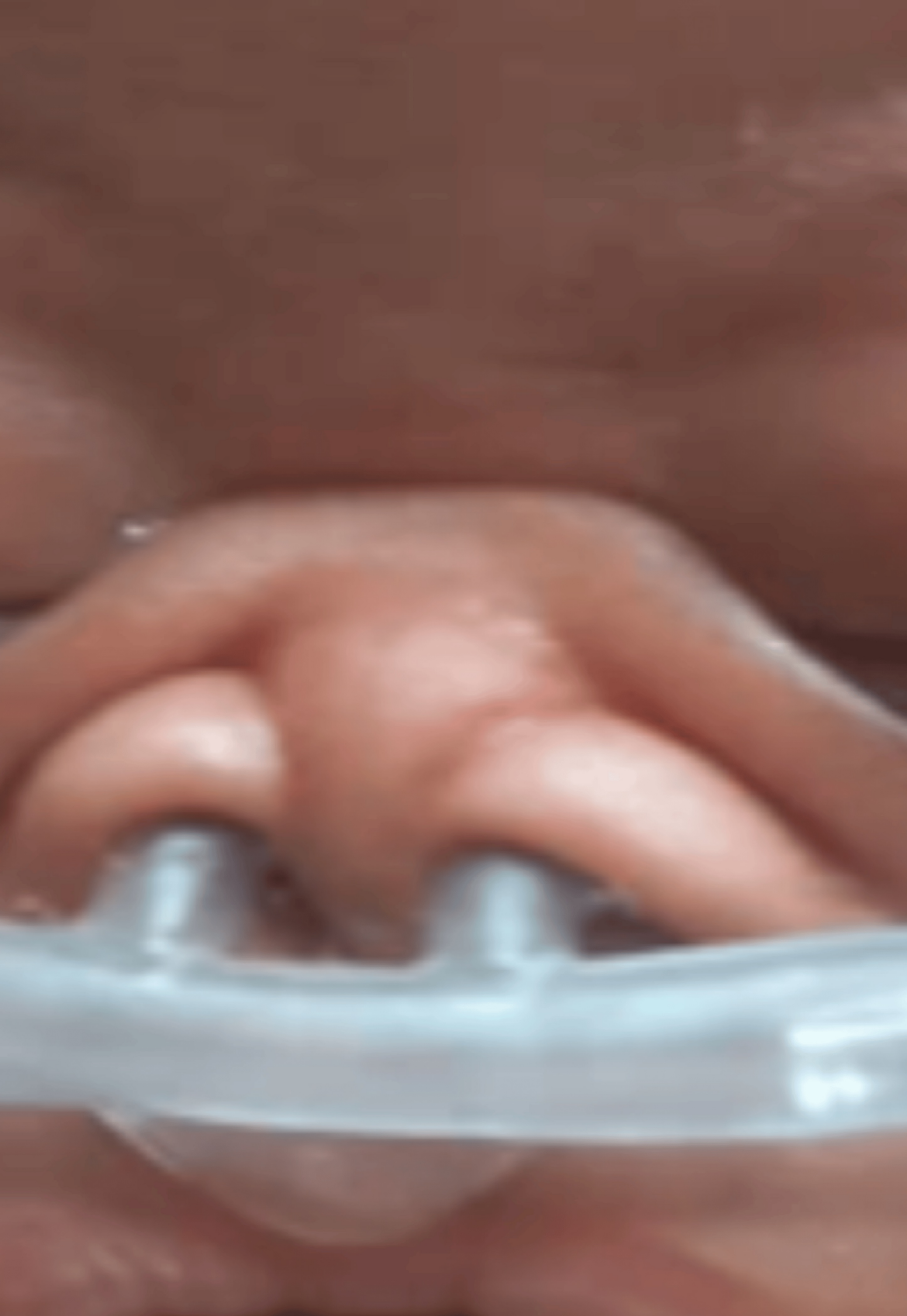
Neonate with a characteristic saddle nose deformity

A baby girl was delivered weighing 2.5 kg, with APGAR scores of 7 and 9 at one and five minutes, respectively. She did not cry immediately and was noted to have respiratory distress at birth. Oxygen therapy via nasal prongs was administered during the first 24 hours of life. Physical examination revealed a saddle nose deformity as shown in Figure 4, and noisy breathing, along with a heart murmur. No other anomalies were observed on physical examination.

Echocardiography revealed a small restrictive mid-muscular ventricular septal defect (Video 2), a small patent ductus arteriosus (PDA) (Video 3), and a small patent foramen ovale (Video 4).

**Video 2:** Small mid-muscular VSD VSD: Ventricular Septal Defect

**Video 3:** Small PDA measuring 0.3 cm PDA: Patent Ductus Arteriosus

**Video 4:** Small PFO PFO: Patent Foramen Ovale

**Figure 5:**
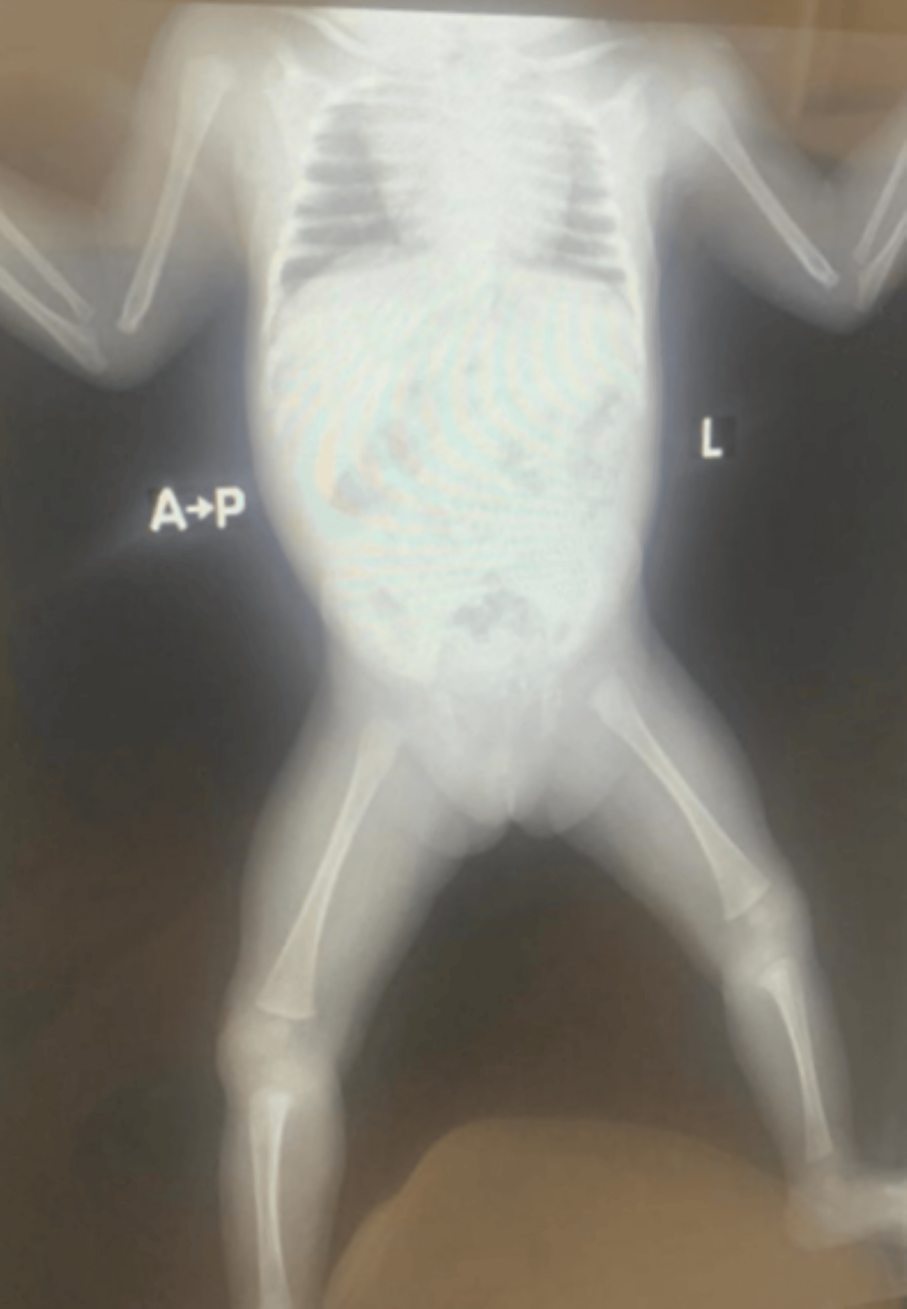
Babygram revealing normal long bones

A follow-up echocardiogram was planned for 12 months later. Further Ear Nose and Throat evaluation confirmed the presence of a saddle nose deformity and laryngotracheomalacia, both of which are being managed conservatively. An X-ray showed no long bone deformities (Figure 5). 

Both the mother and baby remain stable and continue to receive ongoing follow-up care. The baby is growing appropriately and meeting normal developmental milestones.

## Discussion

We presented a case of a female baby born with features of warfarin embryopathy to a mother with an MHV due to RHD, complicated by prosthetic valve thrombosis and cardioembolic stroke. Her pregnancy was diagnosed at 16 weeks of amenorrhea, and she used warfarin at doses equal to or greater than 5mg daily during the first trimester.

A review of warfarin embryopathy cases stated that the minimum criteria to define one is exposure to a coumarin derivative during the first trimester and either the presence of characteristic nasal hypoplasia or stippled epiphyses [6]. The diagnosis of warfarin embryopathy in this case was based on the documented history of maternal warfarin use during the first trimester and the typical facial abnormalities (nasal hypoplasia) in the neonate.

Pregnancy poses significant challenges for women requiring long-term anticoagulation, particularly those with MHVs, due to the ongoing need for anticoagulation therapy to prevent prosthetic valve thrombosis. Pregnancy is itself a prothrombotic state [7,8], increasing maternal risks while also posing potential dangers to fetal development.

The European Society of Cardiology (ESC) Registry of Pregnancy and Cardiac Disease (ROPAC) study reported an increased maternal mortality, fetal loss, and both thrombotic and hemorrhagic complications among women with MHVs compared to those with tissue heart valves or no prosthetic valves [9]. No anticoagulation regimen was found to be superior in all aspects; however, women on vitamin K-antagonists had a lower incidence of mechanical valve thrombosis but poorer fetal outcomes. A systematic review by Chan and colleagues also revealed that warfarin was associated with the lowest risk of maternal thromboembolic events, but this is associated with a higher risk of fetal adverse events [5].

Fetal abnormalities described in warfarin embryopathy are postulated to result from the drug blocking the synthesis of vitamin K-dependent proteins during crucial developmental periods (6 to 14 weeks), including proteins involved in bone and cartilage formation [10,11].

The burden of warfarin embryopathy is stated at about 6% of children born to mothers taking warfarin [5]. A few observational reports suggest that these are more likely to occur at higher warfarin doses (>5mg/day); however, cases of warfarin embryopathy, even at lower doses, have been reported [12]. A study utilizing autopsy of babies with a history of warfarin exposure described these characteristics: shortening and flattening of the nose, deep nasal alar grooves and long bone abnormalities such as epiphyseal stippling, absence of some bones, delayed maturation and dystrophic nails. Some had no bone abnormalities [13]. This case presented with typical facial features but no long bone abnormalities were apparent. Other cases in the literature have reported varying combinations of facial, long bone, and other anomalies including laryngotracheomalacia, with facial anomalies being a consistent finding. Congenital heart defects have also been reported with atrial septal defects and PDA featuring commonly [14,15].

Beyond the described warfarin embryopathy, other fetal complications of in utero warfarin exposure include neurological issues including intracranial hemorrhage, hydrocephalus, developmental delays and cognitive impairment, and congenital heart defects [6,9]. The risk of neurological complications is particularly high with third-trimester exposure, necessitating careful anticoagulation management strategies to balance maternal and fetal safety. In our case, the mother received warfarin for all except the last two weeks of pregnancy during which she received LMWH.

Heparins present a safer option for fetal health as it does not cross the placenta. However, observational studies indicate that heparin use in pregnancy, particularly in high-risk patients with MHVs like in this case, is associated with an increased risk of maternal thrombosis and hemorrhage [6,9]. Given the hypercoagulable state of pregnancy, higher LMWH doses than the standard 1 mg/kg every 12 hours may be necessary to prevent valve thrombosis [12].

According to the American Heart Association (AHA) and American College of Cardiology (ACC) guidelines, anticoagulation management in pregnant women with MHVs should be guided by warfarin dose requirements. If the daily requirement is less than 5mg, warfarin may be continued due to its superior maternal protection against thrombotic events. However, if the daily dose is greater than 5mg, the warfarin may be replaced with LMWH during the first trimester to minimize fetal risk, with rigorous anti-Xa level monitoring. LMWH should be used in the peripartum period, after which patients can resume warfarin postpartum [16]. This case involves a high-risk mother with a history of thrombotic complications related to an MHV. The daily warfarin dose during the first trimester extrapolated from the monthly visits, was equal to or greater than 5mg during the first trimester. The opportunity to adjust anticoagulation strategy was missed because her pregnancy was discovered well after the critical first trimester, and she remained on warfarin until near term, when she was transitioned to LMWH.

This case highlights the unique challenges of managing MHVs in women of reproductive age, particularly in low-income settings, where follow-up is challenging. It underscores the importance of shared decision-making regarding valve selection at the time of heart surgery, contraception, and pre-conception counselling, and the need for ongoing, open communication in case of an unplanned pregnancy. The ultimate goal is to optimize both maternal and fetal safety.

## Conclusions

This case highlights the still present teratogenic risks of warfarin use during pregnancy, particularly its association with warfarin embryopathy. While warfarin remains the most effective anticoagulant for preventing thromboembolic complications in patients with MHVs, its fetal risks necessitate individualized anticoagulation strategies. Careful preconception counseling, close INR monitoring, and appropriate anticoagulation adjustments are crucial in reducing adverse maternal and fetal outcomes. Further research is needed to optimize anticoagulation regimens in pregnancy, particularly in resource-limited settings where RHD among young women remains prevalent.

## References

[1] Santangelo G, Bursi F, Faggiano A (2023). The global burden of valvular heart disease: from clinical epidemiology to management. J Clin Med.

[2] Rwebembera J, Manyilirah W, Zhu ZW (2018). Prevalence and characteristics of primary left-sided valve disease in a cohort of 15,000 patients undergoing echocardiography studies in a tertiary hospital in Uganda. BMC Cardiovasc Disord.

[3] Vahanian A, Beyersdorf F, Praz F (2022). 2021 ESC/EACTS Guidelines for the management of valvular heart disease. Eur Heart J.

[4] Chan V, Malas T, Lapierre H (2011). Reoperation of left heart valve bioprostheses according to age at implantation. Circulation.

[5] Chan WS, Anand S, Ginsberg JS (2000). Anticoagulation of pregnant women with mechanical heart valves: a systematic review of the literature. Arch Intern Med.

[6] Hall JG, Pauli RM, Wilson KM (1980). Maternal and fetal sequelae of anticoagulation during pregnancy,. Am J Med.

[7] Stirling Y, Woolf L, North WR, Segatchian MJ, Meade TW (1984). Haemostasis in normal pregnancy. Thromb Haemost.

[8] Comp PC, Thurnau GR, Welsh J, Esmon CT (1986). Functional and immunologic protein S levels are decreased during pregnancy. Blood.

[9] van Hagen IM, Roos-Hesselink JW, Ruys TP (2015). Pregnancy in women with a mechanical heart valve: data of the European Society of Cardiology Registry of Pregnancy and Cardiac Disease (ROPAC). Circulation.

[10] Menon RK, Gill DS, Thomas M, Kernoff PB, Dandona P (1987). Impaired carboxylation of osteocalcin in warfarin-treated patients. J Clin Endocrinol Metab.

[11] Walfisch A, Koren G (2010). The ‘warfarin window’ in pregnancy: the importance of half-life. J Obstet Gynaecol Canada, vol. 32, no. 10, pp. 988-989.

[12] McLintock C (2014). Thromboembolism in pregnancy: challenges and controversies in the prevention of pregnancy-associated venous thromboembolism and management of anticoagulation in women with mechanical prosthetic heart valves. Best Pract Res Clin Obstet Gynaecol.

[13] Wainwright H, Beighton P (2010). Warfarin embryopathy: fetal manifestations. Virchows Arch.

[14] Starling LD, Sinha A, Boyd D, Furck A (2012). Fetal warfarin syndrome. BMJ Case Rep.

[15] Ferreira S, Costa R, Malveiro D, Vieira F, Tuna M (2018). Warfarin embryopathy: balancing maternal and fetal risks with anticoagulation therapy. Pediatr Neonatol.

[16] Otto CM, Nishimura RA, Bonow RO (2021). 2020 ACC/AHA Guideline for the management of patients with valvular heart disease: a report of the American College of Cardiology/American Heart Association Joint Committee on Clinical Practice Guidelines. Circulation.

